# Association between physical frailty, circadian syndrome and cardiovascular disease among middle-aged and older adults: a longitudinal study

**DOI:** 10.1186/s12877-024-04787-8

**Published:** 2024-02-27

**Authors:** Xinhong Zhu, Linlin Ding, Xiaona Zhang, Heqing Wang, Ningbo Chen

**Affiliations:** 1 School of Nursing, Hubei University of Chinese Medicine, Wuhan, China; 2 Hubei Shizhen Laboratory, Wuhan, China

**Keywords:** Physical frailty, Circadian syndrome, Cardiovascular disease, Middle-aged and older adults

## Abstract

**Background:**

Physical frailty (PF) and circadian syndrome (CircS) are proposed as novel risks for cardiovascular disease (CVD), but little attention is paid to their combined impact on CVD. This study aimed to investigate the association of PF, CircS and CVD in middle-aged and older adults.

**Methods:**

The sample comprised 8512 participants aged at least 45 years from the China Health and Retirement Longitudinal Study (CHARLS) 2011. PF was examined by the physical frailty phenotype scale. CircS was assessed by the components of the International Diabetes Federation (IDF) MetS plus short sleep duration and depression. The cut-off for CircS was set as ≥ 4. CVD was defined as the presence of physician-diagnosed heart disease and/or stroke. A total of 6176 participants without CVD recruited from CHARLS 2011 and were followed up in 2018.

**Results:**

The prevalence of CVD in total populations, neither CircS or PF, PF alone, CircS alone and both CircS and PF were 13.0%, 7.4%, 15.5%, 17.4%, and 30.2%, respectively. CircS was more likely to be PF [OR (95%CI): 2.070 (1.732 ∼ 2.472)] than those without CircS. Both CircS alone [OR (95% CI): 1.954 (1.663 ∼ 2.296)], and coexisting CircS and PF [3.508 (2.739 ∼ 4.494)] were associated with CVD. Longitudinal analysis showed that individuals with both CircS and PF (HR: 1.716, 95%CI: 1.314 ∼ 2.240) and CircS alone [1.520 (1.331 ∼ 1.737)] were more likely to have new onset CVD than neither CircS or PF peers.

**Conclusion:**

PF and CircS together are associated with higher CVD risk, which provided new evidence for a strong relation that warrants attention to assessing PF and CircS and in community to promote healthy aging.

**Supplementary Information:**

The online version contains supplementary material available at 10.1186/s12877-024-04787-8.

## Introduction

Frailty, characterized by loss of functional reserve and diminished resistance to stressors, is multidimensional syndrome that encompasses physical, social and cognitive dimensions [1, 2]. Among them, the physical frailty (PF) is the most commonly investigated component, which is defined based on the phenotypic criteria of weakness, slowness, low physical activity, weight loss, and exhaustion [1]. PF has been shown in many studies to predict fall, disability, multimorbidity and mortality [3–5]. Cardiovascular disease (CVD), the leading cause of both death and premature death in China, is the major threat to health and independence of people [6]. Findings has revealed that frailty affects almost half of patients with heart failure, and the overall estimated prevalence of PF in heart failure is 42.9% [7]. Additionally, PF is also implicated as a causative and prognostic factor in patients with CVD [8]. In the cross-sectional survey, stroke and heart disease were all independently associated with higher physical frailty among community-dwelling Chinese elderly [9]. Besides, compared with the non-frail people, longitudinal evidence showed that physical frailty increased incident rate of overall cardiometabolic diseases including hypertension, diabetes, and cardiovascular diseases [10, 11].

Recently, circadian dysfunction has been proposed as an important underlying etiological factor for the metabolic syndrome (MetS) [12]. The concept of the circadian syndrome (CircS) is proposed based on the evidence that circadian dysfunction is a potential driver of many chronic disorders including obesity [13], diabetes [14], kidney disease [15], and non-alcoholic fatty liver disease (NAFLD) [16]. Disturbances in sleep patterns and depressive symptoms can cause circadian rhythm disruption [17, 18]. Therefore, CircS was proposed as a novel risk cluster for CVD based on MetS plus short sleep duration and depressive symptoms [19, 20]. In addition, epidemiological cohort studies established short sleep duration and depression as risk factors for developing CVD and CVD mortality [21–23]. Both CircS and PF are associated with increased incident CVD, yet the effect of coexisting these two conditions on CVD events has not been elucidated.

Given the rapid aging population in China and the rise in physical frailty and MetS [24, 25], understanding these co-dynamics will likely be of increasing importance. The relationship between MetS and frailty may be bidirectional and share the common mechanisms [26, 27]. Older adults with MetS are more likely to be frailty [28]. Moreover, individuals with short sleep duration or depressive symptoms are likely to exhibit PF [29, 30]. Additionally, sarcopenia, as the main component of PF, shared extensive clinical similarities and biomarkers with PF [31]. The prevalence of sarcopenia was higher in those with MetS, and increased muscle mass strongly inhibits the development of MetS [32, 33]. Furthermore, evidence showed that patients with MetS and sarcopenia at the same time had a higher risk of several adverse health events than those with either MetS alone or sarcopenia alone [34]. Collectively, coexisting these conditions may result in worse health outcome than either condition alone. Given sarcopenia as the biological substrate of PF [35], the association of CircS, PF and CVD need to be explored further.

For now, the contribution of individual PF and CircS status to incident CVD is still unknown. In this study, we explored associations between PF and CircS, individually or in combination, with CVD in middle-aged and older adults participating in the Chinese Health and Retirement Longitudinal Study (CHARLS), and determined if coexistence of both PF and CircS carries a higher likelihood of CVD.

## Methods

### Study population

The CHARLS established in 2011, is an ongoing nationally representative longitudinal survey in China [36]. In brief, CHARLS collects high-quality data from a nationally representative sample of the Chinese population aged at least 45 years older, selected using multistage stratified probability-proportionate-to-size sampling, through one-on-one interviews using a structured questionnaire. The study has been conducted in 28 provinces and covers 150 counties/districts and 450 villages/urban communities across China [36]. All participants were followed up every 2 years after the baseline survey.

**Fig. 1:**
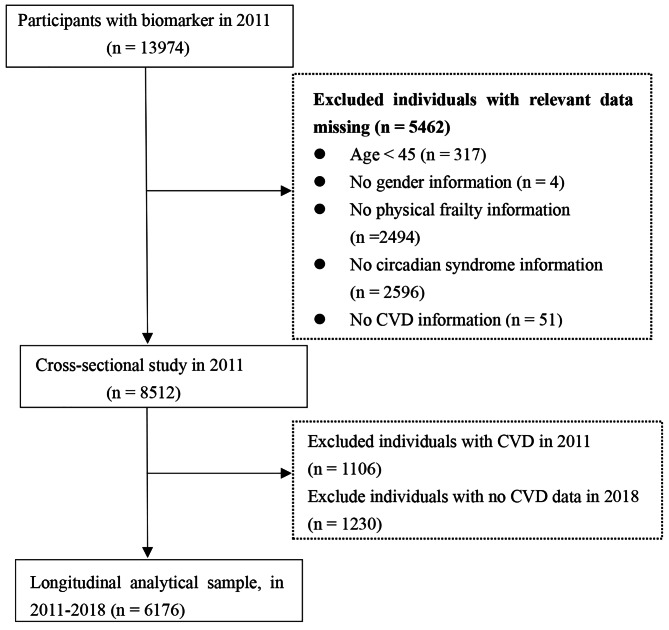
Flow diagram for participants included in the study

In the present study, we retrospectively analyzed data from the CHARLS 2011 and 2018. The exclusion criteria for the present study were as follows: (1) individuals aged less than 45 years old; (2) missing data on physical frailty status; (3) no information about age and sex; (4) lack of CircS information; and (5) lack of CVD information. Our final analytic sample included 6176 persons, who had no CVD in the CHARLS 2011 and were fully followed up in 2018. The detailed selection process is presented in Fig. 1.

### Assessment of physical frailty

PF was measured by a modified version of the Fried physical frailty phenotype approach [1], which has been developed and validated in CHARLS [37]. Five items are included: shrinking, weakness, slowness, low physical activity, and exhaustion. Shrinking was established as self-reported loss of 5 or more kg in the previous year or having a body mass index (BMI) of 18.5 kg/m^2^ or less. Weakness was defined using a handgrip strength test, which was measured in the dominant hand and nondominant hand, with the participant squeezing a YuejianTM WL-1000 dynamometer (Nantong Yuejian Physical Measurement Instrument Co., Ltd., Nantong, China) as hard as possible [36]. The cut-off points for low grip strength for men and women were < 28 and < 18 kg, respectively. Slowness was determined using a 2.5 m gait speed or the chair stand test described by Wu et al [38]. Low physical activity was determined if participants self-reported that they did not walk 10 or more minutes continuously during a usual week. Exhaustion was determined according to two questions from the 10-item Center for Epidemiological Studies Depression Scale (CESD-10): “I felt everything I did was an effort” and “I could not get going”. If the subjects responded “sometimes or half of the time (3–4 days)” or “most of the time (5–7 days) to either item, they were categorized as having self-reported exhaustion. Participants who met three or more criteria were defined as having physical frailty; otherwise, they were considered as having no physical frailty.

### Assessment of CircS

Fasting blood samples were collected and stored at-80 °C for further determining the triglycerides, glucose, C-reactive protein (CRP), uric acid, creatinine, high-density lipoprotein (HDL) cholesterol, and hemoglobin using enzymatic colorimetric tests.

Depression symptoms was evaluated using the ten-item Center for Epidemiologic Studies Depression Scale (CESD-10). The cut-off score of ≥ 10 was used to identify the respondents who had depressive symptoms significantly.

CircS was included abdominal obesity (waist circumference ≥ 85 cm in men, and ≥ 80 cm in women), hypertension (systolic blood pressure ≥ 130 mmHg and/or diastolic blood pressure ≥ 85 mmHg, or drug treatment for hypertension), hyperglycemia (≥ 100 mg dl^-1^ or drug treatment for elevated glucose), high triglycerides (≥ 150 mg dl^-1^ or drug treatment for high triglycerides), low HDL cholesterol (< 40 mg dl^-1^ or drug treatment for low HDL cholesterol), depression and short sleep duration (< 6 h day^-1^), which has been developed and validated in CHARLS [19, 39]. Participants who met four or more criteria were defined as having CircS; otherwise, they were considered as having no CircS.

### Assessment of CVD events

The study outcome was CVD events, including heart disease and stroke. Similar to previous study [40, 41], CVD was assessed by the following questions: “Have you been told by a doctor that you have been diagnosed with a heart attack, angina, coronary heart disease, heart failure, or other heart problems?” or “Have you been told by a doctor that you have been diagnosed with a stroke?”. Participants who reported heart disease or stroke during the follow-up period were defined as having incident CVD. The diagnosis date of incident heart disease or stroke cases was determined by the date between the time of the survey that first recorded heart disease/stroke patients and the time of the previous round of survey.

### Covariates

At baseline, trained interviewers collected information on socio-demographic status and health-related factors using a structured questionnaire. Sociodemographic variables included age, gender, education (illiterate, primary school or below, junior high school or above), marital status (married and others), and residence (rural, urban). Health-related factors included BMI, fall, smoking and drinking status (yes or no), wearing glasses (no, yes or blindness), self-reported physician-diagnosed chronic kidney disease, and chronic lung diseases. BMI was divided into three groups: normal weight (BMI = 18.5 kg/m^2^ to 24 kg/m^2^), underweight (BMI < 18.5 kg/m^2^), and overweight or obesity (BMI ≥ 24 kg/m^2^).

### Statistical analysis

Data are presented as the mean ± standard deviation (SD) and percentages for categorical variables. Baseline characteristics was summarized based on PF and CircS status and compared using the chi-squared test or analysis of variance. Logistic regression analysis was used to estimate the association between CircS and PF in the Table 2. Logistic regression analysis was conducted with CVD as the outcome variable, and PF and CircS status as the exposure variables in the Table 3. Cox proportional hazards models were used to estimate the relationship between baseline PF and CircS status and incident CVD. The cox proportional hazards models’ parameter estimates were expressed as the hazard ratios (HRs) and the 95% confidence intervals (95% CIs). A *p*-value < 0.05 was considered to indicate statistical significance.

In the cross-sectional and longitudinal analyses, two models were estimated: in Model 1, age and sex was adjusted; in Model 2, age, gender, residence, marital status, educational level, smoking status, drinking status, BMI, fall (s), wearing glass, history of chronic kidney disease, and lung diseases were adjusted.

## Results

### Baseline characteristics

Table 1 shows that the mean (SD) age of the participants was 58.63 (9.18), and 4451 (52.3%) were female. The prevalence of PF alone, CircS alone and coexisting PF and CircS was 6.0%, 36.9% and 6.1%, respectively. Compared to neither PF or CircS, persons with coexisting PF and CircS were more likely to be older, unmarried, be female and less educated, and live in rural setting.

**Table 1:** Baseline characteristics of all participants (*n* = 8512)

Characteristics	Neither CircS or PF (*n* = 4340)	PF only (*n* = 509)	CircS only (*n* = 3144)	Both CircS and PF (*n* = 519)	P
Age, y	56.93 ± 8.55	67.55 ± 8.98	58.17 ± 8.51	66.89 ± 9.10	< 0.001
Male	2383 (54.9)	296 (58.2)	1199 (38.1)	183 (35.3)	< 0.001
Married (vs. others)	3952 (91.1)	412 (80.9)	2792 (88.8)	381 (73.4)	< 0.001
Rural (vs. urban)	2843 (65.5)	402 (79.01)	1751 (55.7)	356 (68.6)	< 0.001
Education level					
Illiterate	983 (22.6)	217 (42.7)	797 (25.3)	250 (48.2)	< 0.001
Primary school or below	1795 (41.4)	233 (45.9)	1247 (39.7)	213 (41.0)	
Junior high school or above	1562 (36.0)	58 (11.4)	1100 (35.0)	56 (10.8)	
Smoking	1607 (37.0)	191 (37.5)	719 (22.9)	118 (22.7)	< 0.001
Drinking	1701 (39.2)	153 (30.1)	905 (28.8)	109 (21.0)	< 0.001
BMI, kg/m^2^	22.7 ± 3.36	19.72 ± 3.27	25.56 ± 3.68	23.34 ± 4.56	< 0.001
BMI category, n (%)					
Underweight	159 (3.7)	234 (46.5)	20 (0.6)	82 (16.2)	< 0.001
Normal weight	2927 (67.8)	223 (44.3)	1078 (34.4)	209 (41.4)	
Overweight or obesity	1232 (28.5)	46 (9.2)	2034 (64.9)	214 (42.4)	
Wearing glasses, n (%)					
No	3884 (89.5)	460 (90.4)	2739 (87.1)	449 (86.7)	< 0.001
Yes	432 (10.0)	48 (9.4)	389 (12.4)	63 (12.2)	
Blindness	24 (0.6)	1 (0.2)	16 (0.5)	6 (1.2)	
Fall (s) (last year)	535 (12.3)	133 (26.2)	493 (15.7)	157 (30.3)	< 0.001
Chronic disease					
Chronic kidney disease	244 (5.6)	47 (9.3)	225 (7.2)	51 (9.8)	< 0.001
Chronic lung disease	353 (8.2)	111 (21.9)	284 (9.0)	87 (16.9)	< 0.001
Hypertension	467 (10.8)	76 (15.0)	1286 (41.1)	245 (47.4)	< 0.001
Diabetes	96 (2.2)	22 (4.3)	347 (11.1)	81 (15.7)	< 0.001

### Cross-sectional associations of PF, CircS and CVD

In the cross-sectional study, the prevalence of CVD in total populations, neither CircS or PF, PF alone, CircS alone and coexisting PF and CircS were 13.0%, 7.4%, 15.5%, 17.4%, and 30.2%, respectively. As depicted in Table 2, CircS was more likely to be PF [OR (95%CI): 2.070 (1.732 ∼ 2.472)] than those without CircS. Similar results were observed both in male and female (Table S1). After adjustment for socio-demographic characteristics and health-related factors, coexisting PF and CircS was significantly associated with CVD [OR (95%CI): 3.508 (2.739 ∼ 4.494)], heart disease [OR (95%CI): 3.218 (2.481 ∼ 4.173)] and stroke [4.675 (2.729 ∼ 8.007)] (Table 3). However, PF alone was not significantly associated with CVD events in male (Table S2).

**Table 2:** The relationship between CircS and PF in the cross-sectional analysis (*n* = 8512)

Variable	OR (95%CI)	
	Model1 ^a^	Model 2 ^b^
CircS	1.214 (1.052 ∼ 1.401) *	2.070 (1.732 ∼ 2.472) **

** *P* < 0.001; * *P* < 0.05

^a^: Model 1 was adjusted for age and gender

^b^: Model 2 was adjusted for age, gender, residence, marital status, educational level, smoking status, drinking status, BMI, fall (s), wearing glass, history of chronic kidney disease and chronic lung diseases

**Table 3:** ORs and 95% CIs of CVD and its components by CircS and PF in the cross-sectional analysis (*n* = 8512)

Outcome	Case	OR (95%CI)	
		Model1 ^a^	Model 2 ^b^
CVD	1106		
Neither CircS or PF	322	Reference	Reference
PF only	79	1.671 (1.268 ∼ 2.201) **	1.830 (1.336 ∼ 2.508) **
CircS only	548	2.420 (2.084 ∼ 2.809) **	1.954 (1.663 ∼ 2.296) **
Both CircS and PF	157	3.782 (3.003 ∼ 4.765) **	3.508 (2.739 ∼ 4.494) **
Heart disease	987		
Neither CircS or PF	289	Reference	Reference
PF only	67	1.568 (1.169 ∼ 2.104) *	1.836 (1.319 ∼ 2.557) **
CircS only	497	2.404 (2.056 ∼ 2.810) **	1.969 (1.663 ∼ 2.331) **
Both CircS and PF	134	3.422 (2.685 ∼ 4.361) **	3.218 (2.481 ∼ 4.173) **
Stroke	163		
Neither CircS or PF	39	Reference	Reference
PF only	14	2.259 (1.190 ∼ 4.289) *	1.973 (0.919 ∼ 4.237)
CircS only	79	2.839 (1.918 ∼ 4.203) **	2.162 (1.426 ∼ 3.279) **
Both CircS and PF	31	5.398 (3.236 ∼ 9.006) **	4.675 (2.729 ∼ 8.007) *

** *P* < 0.001; * *P* < 0.05

^a^: Model 1 was adjusted for age and gender

^b^: Model 2 was adjusted for age, gender, residence, marital status, educational level, smoking status, drinking status, BMI, fall (s), wearing glass, history of chronic kidney disease and chronic lung diseases

### Longitudinal association between baseline PF and CircS status, and incident CVD at follow-up, 2011–2018

As depicted in Table 4,1335 cases (16.5%) with incident CVD events were identified. After adjusting covariates in Model 2, CircS alone [HR (95%CI): 1.520 (1.331 ∼ 1.737)], and coexisting PF and CircS [HR (95%CI): 1.716 (1.314 ∼ 2.240)] had higher risk of CVD compared with either PF or CircS (Table 4). However, PF alone was not significantly associated with the incident CVD. Furthermore, PF alone and coexisting PF and CircS did not predict the incident CVD and its components in male (Table S3).

**Table 4:** Incidence of CVD according to baseline CircS and PF, 2011 − 2018 (*n* = 6176)

Outcome	Case	HR (95%CI)	
		Model1 ^a^	Model 2 ^b^
CVD	1446		
Neither CircS or PF	481	Reference	Reference
PF only	58	1.110 (0.839 ∼ 1.467)	1.262 (0.927 ∼ 1.717)
CircS only	537	1.680 (1.482 ∼ 1.905) **	1.520 (1.331 ∼ 1.737) **
Both CircS and PF	70	1.709 (1.318 ∼ 2.216) **	1.716 (1.314 ∼ 2.240) **
Heart disease	857		
Neither CircS or PF	368	Reference	Reference
PF only	41	1.048 (0.753 ∼ 1.459)	1.175 (0.816 ∼ 1.693)
CircS only	392	1.567 (1.355 ∼ 1.811) **	1.386 (1.189 ∼ 1.617) **
Both CircS and PF	56	1.756 (1.311 ∼ 2.352) **	1.744 (1.291 ∼ 2.355) **
Stroke	388		
Neither CircS or PF	145	Reference	Reference
PF only	20	1.175 (0.728 ∼ 1.897)	1.461 (0.868 ∼ 2.458)
CircS only	195	2.149 (1.727 ∼ 2.675) **	1.958 (1.552 ∼ 2.470) **
Both CircS and PF	28	2.335 (1.534 ∼ 3.554) **	2.302 (1.491 ∼ 3.554) **

** *P* < 0.001

a: Model 1 was adjusted for age and gender

b: Model 2 was adjusted for age, gender, residence, marital status, educational level, smoking status, drinking status, BMI, fall (s), wearing glass, history of chronic kidney disease and chronic lung diseases

## Discussion

In the present study, we found that PF and CircS together was independently and positively associated with CVD in the cross-sectional analysis. Importantly, longitudinal analysis confirmed that individuals with combined PF and CircS were at higher risk of new onset CVD among middle-aged and older adults compared to persons with CircS alone.

In this study, CircS are common among middle aged and older people. The prevalence of CircS was 43.0%, which is higher than that reported by Shi et al. (40.9%) in the context of adults aged above 20 years in the US [20]. Due to circadian disruption acting as an important underlying etiological factor for the MetS, the CircS is proposed [12]. Evidence showed that the pooled prevalence of MetS increased with age [42]. Additionally, the prevalence of CircS is higher than reported by Shi et al. (39.0%) from the same cohort [19], which may be attributed to different inclusion criteria. Physical frailty is geriatric syndrome and significantly associated with MetS [27], which may lead to an increase in morbidity.

In the line with previous studies [19, 20], the CircS is a significant and stronger predictor for CVD. In a meta-analysis of 87 studies, Mottillo et al. found that MetS had a relative risk of 2.35 (95%CI 2.02–2.73) for CVD [43]. Two additional components, short sleep and depression, were added into the components of MetS to construct the CircS [12]. Additionally, previous studies revealed that short sleep duration and depression symptom were identified as risk factors for developing CVD and CVD mortality [21–23]. A meta-analysis study encompassing 54,250 patients with a mean weighted follow-up of 6.2 years, showed that frailty has been implicated as a causative and prognostic factor in patients with CVD [44]. Besides, as frailty phenotype, physical frailty was causally related to high risk of incident CVD in UK individuals [10]. However, in this study, physical frailty alone was significantly associated with CVD, but did not predict the incident CVD. Evidence showed that the longitudinal associations between possible sarcopenia, sarcopenia, and heart diseases were not significant after further adjusting for metabolic biomarkers among the subpopulations of 9753 subjects with metabolic biomarkers measurements [40]. In addition, oxidative stress and systemic inflammation may act as potential contributors to frailty and CVD, and to explain a possible pathogenic relationship between the latter two conditions [45]. Abnormal metabolic biomarkers (e.g. TG, HDL cholesterol, hyperglycemia) may play important roles in the metabolic homeostasis. Therefore, physical frailty with metabolic health may not be able to predict incident CVD. In this study, physical frailty in male was not associated with CVD events. In community dwelling populations aged over 65 years, women are more likely to be frail and to have a greater burden of frailty than men of the same age [46, 47].

In the present study, coexisting physical frailty and CircS had higher risk of incident CVD than either condition alone. Evidence showed that MetS and frailty shared some common mechanisms including insulin resistance, arteriosclerosis, chronic inflammation, oxidative stress, and mitochondrial dysfunction [26]. MetS in those aged over 50 years old was found to be associated with an increased likelihood of incident frailty over a 4-year period [48]. A meta-analysis points to a reciprocal interaction between depression and frailty in older adults [49]. Meanwhile, previous study showed that short sleep duration significantly elevated the risk for frailty [50]. In this study, CircS was positively associated with PF. Furthermore, previous study had revealed that patients with MetS and sarcopenia at the same time had a higher risk of several adverse health events than those with either MetS alone or sarcopenia alone [34]. In general, CircS increased the risk of incident CVD, and presence of physical frailty elevated this risk.

There are several limitations in the current study. First, the exclusion of participants with missing data on CircS and PF, and lost to follow-up would have selection bias and resulted in limited sample size for outcome. Second, this study used observational data, which may have biased the observed relationships by introducing confounding factors. To reduce such bias, we considered as many related factors as possible in the analysis. Third, consistent with other previous studies [40, 51], the diagnosis of CVD was self-reported physician-diagnosed. Medical records were not available in the CHARLS; thus, the use of self-reported measures of CVD may also exhibit some degree of bias. However, in the English Longitudinal Study of Ageing 77.5% of self-reported incident coronary heart disease were confirmed by medical records [52]. Forth, the association between CircS, PF and specific heart disease, such as heart attack, coronary heart disease and heart failure, could not be detected because of the lack of detailed classification of CVD. These important questions should be taken into account in subsequent studies.

In conclusion, this study demonstrated CircS was associated with physical frailty in community-dwelling middle-aged and older individuals. The presence of coexisting CircS and physical frailty were much more likely to develop new onset CVD than either condition alone. Collectively, our findings provided the new evidence for an association of physical frailty, CircS and CVD, and that further research is required to elucidate the underlying causal mechanism for coexisting these two conditions contributions to CVD. The study findings suggested the importance of screening for physical frailty and CircS in middle-aged and older people. Early detection of physical frailty and CircS may allow for optimization of treatment, resulting in better health outcomes.

### Electronic supplementary material

Below is the link to the electronic supplementary material.

Supplementary Material 1

## Data Availability

The data used in the study are accessible to be downloaded publicly at https://charls.charlsdata.com/pages/data/111/zh-cn.html.
