# Supplementary material for: Association between physical frailty, circadian syndrome and cardiovascular disease among middle-aged and older adults: a longitudinal study
Source: BMC Geriatr. 2024 Feb 27;24:199. doi: 10.1186/s12877-024-04787-8 (PMC10900721; doi:10.1186/s12877-024-04787-8)
Supplement: Supplementary file 1 — Supplementary Material 1 [file 12877_2024_4787_MOESM1_ESM.docx]

Table S1 Subgroup analysis of the relationship between CircS and PF by sex (n = 8512)

| Variable | 95%OR | | | |
| --- | --- | --- | --- | --- |
|  | Male | | Female | |
|  | Model1 ^a^ | Model 2 ^b^ | Model1 ^a^ | Model 2 ^b^ |
| CircS | 1.244 (1.007~1.538) * | 2.103 (1.617~2.737) ** | 1.208 (0.994~1.469) | 2.100 (1.644~2.682) ** |

** *P* < 0.001; * *P* < 0.05

^a^: Model 1 was adjusted for age

^b^: Model 2 was adjusted for age, residence, marital status, educational level, smoking status, drinking status, BMI, fall (s), wearing glass, history of chronic kidney disease and chronic lung diseases.

Table S2 Subgroup analysis of relationship of CircS, PF and CVD events by sex (n = 8512)

| Outcome | 95%OR | | | |
| --- | --- | --- | --- | --- |
|  | Male | | Female | |
|  | Model1 ^a^ | Model 2 ^b^ | Model1 ^a^ | Model 2 ^b^ |
| CVD |  |  |  |  |
| Neither CircS or PF | Reference | Reference | Reference | Reference |
| PF only | 1.292 (0.870~1.919) | 1.296 (0.817~2.056) | 2.134 (1.450~3.141) ** | 2.499 (1.613~3.872) ** |
| CircS only | 2.577 (2.054~3.233) ** | 1.927 (1.499~2.479) ** | 2.368 (1.940~2.891) ** | 1.993 (1.612~2.456) ** |
| Both CircS and PF | 4.247 (2.952~6.111) ** | 3.684 (2.474~5.486) ** | 3.645 (2.699~4.923) ** | 3.545 (2.572~4.886) ** |
| Heart disease |  |  |  |  |
| Neither CircS or PF | Reference | Reference | Reference | Reference |
| PF only | 1.203 (0.787~1.839) | 1.293 (0.792~2.110) | 2.005 (1.332~3.017) * | 2.507 (1.589~3.955) ** |
| CircS only | 2.518 (1.984~3.195) ** | 1.934 (1.484~2.520) ** | 2.393 (1.943~2.947) ** | 2.030 (1.626~2.535) ** |
| Both CircS and PF | 3.561 (2.419~5.244) ** | 3.206 (2.098~4.899) ** | 3.470 (2.536~4.748) ** | 3.374 (2.415~4.714) ** |
| Stroke |  |  |  |  |
| Neither CircS or PF | Reference | Reference | Reference | Reference |
| PF only | 1.980 (0.841~4.660) | 1.910 (0.697~5.237) | 2.568 (0.976~6.755) | 2.038 (0.616~6.741) |
| CircS only | 3.583 (2.086~6.156) ** | 2.515 (1.396~4.532) * | 2.222 (1.267~3.897) * | 1.817 (1.013~3.260) * |
| Both CircS and PF | 6.263 (3.037~12.913) ** | 5.170 (2.362~11.316) ** | 4.622 (2.247~9.508) ** | 4.182 (1.971~8.871) ** |

** *P* < 0.001; * *P* < 0.05

^a^: Model 1 was adjusted for age

^b^: Model 2 was adjusted for age, residence, marital status, educational level, smoking status, drinking status, BMI, fall (s), wearing glass, history of chronic kidney disease and chronic lung diseases.

Table S3 Subgroup analysis of longitudinal association of CircS, PF and CVD events by sex in (n = 6176)

| Outcome | 95%HR | | | |
| --- | --- | --- | --- | --- |
|  | Male | | Female | |
|  | Model1 ^a^ | Model 2 ^b^ | Model1 ^a^ | Model 2 ^b^ |
| CVD |  |  |  |  |
| Neither CircS or PF | Reference | Reference | Reference | Reference |
| PF only | 1.056 (0.719~1.550) | 1.232 (0.804~1.887) | 1.174 (0.780~1.776) | 1.321 (0.843~2.071) |
| CircS only | 1.582 (1.310~1.911) ** | 1.372 (1.116~1.687) ** | 1.766 (1.490~2.094) ** | 1.630 (1.363~1.948) ** |
| Both CircS and PF | 1.526 (0.939~2.477) | 1.523 (0.931~2.493) | 1.835 (1.339~2.514) ** | 1.842 (1.331~2.550) ** |
| Heart disease |  |  |  |  |
| Neither CircS or PF | Reference | Reference | Reference | Reference |
| PF only | 0.887 (0.546~1.441) | 1.032 (0.601~1.771) | 1.226 (0.779~1.929) | 1.360 (0.826~2.240) |
| CircS only | 1.368 (1.086~1.742) ** | 1.120 (0.871~1.441) | 1.726 (1.426~2.089) ** | 1.578 (1.292~1.927) ** |
| Both CircS and PF | 1.510 (0.835~2.672) | 1.493 (0.835~2.667) | 1.952 (1.378~2.765) ** | 1.951 (1.360~2.798) ** |
| Stroke |  |  |  |  |
| Neither CircS or PF | Reference | Reference | Reference | Reference |
| PF only | 1.340 (0.750~2.397) | 1.695 (0.901~3.187) | 0.940 (0.399~2.217) | 1.145 (0.453~2.895) |
| CircS only | 2.093 (1.560~2.810) ** | 1.917 (1.390~2.645) ** | 2.186 (1.568~3.048) ** | 1.984 (1.404~2.804) ** |
| Both CircS and PF | 1.905 (0.914~3.968) | 1.897 (0.899~4.003) | 2.525 (1.475~4.323) * | 2.470 (1.410~4.329) ** |

** *P* < 0.001; * *P* < 0.05

^a^: Model 1 was adjusted for age

^b^: Model 2 was adjusted for age, residence, marital status, educational level, smoking status, drinking status, BMI, fall (s), wearing glass, history of chronic kidney disease and chronic lung diseases.
